# Responses of Marine Diatom-Dinoflagellate Competition to Multiple Environmental Drivers: Abundance, Elemental, and Biochemical Aspects

**DOI:** 10.3389/fmicb.2021.731786

**Published:** 2021-08-30

**Authors:** Rong Bi, Zhong Cao, Stefanie M. H. Ismar-Rebitz, Ulrich Sommer, Hailong Zhang, Yang Ding, Meixun Zhao

**Affiliations:** ^1^Frontiers Science Center for Deep Ocean Multispheres and Earth System, and Key Laboratory of Marine Chemistry Theory and Technology, Ocean University of China, Ministry of Education, Qingdao, China; ^2^Laboratory for Marine Ecology and Environmental Science, Qingdao National Laboratory for Marine Science and Technology, Qingdao, China; ^3^Marine Ecology, GEOMAR Helmholtz Centre for Ocean Research Kiel, Kiel, Germany

**Keywords:** *Phaeodactylum tricornutum*, *Prorocentrum minimum*, elemental stoichiometry, fatty acids, sterols, temperature, nutrients

## Abstract

Ocean-related global change has strongly affected the competition between key marine phytoplankton groups, such as diatoms and dinoflagellates, especially with the deleterious consequency of the increasing occurrence of harmful algal blooms. The dominance of diatoms generally shifts toward that of dinoflagellates in response to increasing temperature and reduced nutrient availability; however, contradictory findings have also been observed in certain sea areas. A key challenge in ecology and biogeochemistry is to quantitatively determine the effects of multiple environmental factors on the diatom-dinoflagellate community and the related changes in elemental and biochemical composition. Here, we test the interplay between temperature, nutrient concentrations and their ratios on marine diatom-dinoflagellate competition and chemical composition using bi-algal competition experiments. The ubiquitous diatom *Phaeodactylum tricornutum* and dinoflagellate *Prorocentrum minimum* were cultivated semi-continuously, provided with different N and P concentrations (three different levels) and ratios (10:1, 24:1, and 63:1 molar ratios) under three temperatures (12, 18, and 24°C). The responses of diatom-dinoflagellate competition were analyzed by a Lotka-Volterra model and quantified by generalized linear mixed models (GLMMs) and generalized additive models (GAMs). The changes in nutrient concentrations significantly affected diatom-dinoflagellate competition, causing a competitive superiority of the diatoms at high nutrient concentrations, independent of temperature and N:P supply ratios. Interestingly, the effect amplitude of nutrient concentrations varied with different temperatures, showing a switch back toward a competitive superiority of the dinoflagellates at the highest temperature and at very high nutrient concentrations. The ratios of particulate organic nitrogen to phosphorus showed significant negative correlations with increasing diatoms/dinoflagellates ratios, while lipid biomarkers (fatty acids and sterols) correlated positively with increasing diatoms/dinoflagellates ratios over the entire ranges of temperature, N and P concentrations and N:P ratios. Our results indicate that the integration of phytoplankton community structure and chemical composition provides an important step forward to quantitatively understand and predict how phytoplankton community changes affect ecosystem functions and biogeochemical cycles in the ocean.

## Introduction

The changes in hydrological conditions and climate warming have strongly influenced phytoplankton community composition, which consequently affects marine food webs and biogeochemical cycles as different phytoplankton groups have varying contributions to the ocean’s biological pump (Falkowski and Oliver, 2007). It has been increasingly realized that the pivotal roles of key phytoplankton groups in marine ecosystems relate with distinctive chemical composition of each group (Dalsgaard et al., 2003; Volkman, 2003; Weber and Deutsch, 2010; Shemi et al., 2016; Jo et al., 2021). C:N:P stoichiometry in marine phytoplankton primarily reflects the evolutionary inheritance, e.g., lower N:P molar ratios in diatoms compared to dinoflagellates (Quigg et al., 2003). Determining biologically and abiotically driven variability in phytoplankton C:N:P stoichiometry (particulate organic carbon to nitrogen (POC/PON) and particulate organic nitrogen to phosphorus (PON/POP) hereafter) is of critical importance for understanding ocean biogeochemical cycles and their interactions with environmental changes (Tyrrell, 1999; Martiny et al., 2013; Lee et al., 2021). Equally important is biochemical composition in phytoplankton. Certain phytoplankton-produced biomolecules (biomarkers), such as fatty acids (FAs) and sterols have been applied as trophic markers for tracing food web structures (Dalsgaard et al., 2003; Martin-Creuzburg and von Elert, 2009; Ruess and Müller-Navarra, 2019; Kohlbach et al., 2021) and as proxies for reconstructing phytoplankton community structure in the past and present-day ocean (Schubert et al., 1998; Zimmerman and Canuel, 2002; Wu et al., 2016; Wang et al., 2019). Ocean-related global change has significant effects on the chemical composition of phytoplankton (Guschina and Harwood, 2009; Hixson and Arts, 2016; Ding et al., 2019; Bi et al., 2020), e.g., showing up to a 83% increase in stoichiometric P:C in response to warming and a 76% increase in carbon-normalized contents of polyunsaturated fatty acids (PUFAs) in response to nutrient deficiency (Bi et al., 2017). Despite this increasing awareness of strong variations in the chemical composition of phytoplankton, little is known about the quantitative relationship between marine phytoplankton community structure and its chemical composition in the changing environments.

Diatoms and dinoflagellates are major phytoplankton groups in the ocean. Diatoms contribute as much as 40% of the total annual organic carbon production in the sea and occupy the base of the simple, ecologically efficient food web which is typical at high nutrient regimes with rapid changes of light conditions (Sommer et al., 2002; Armbrust et al., 2004). Adaptation of diatoms to such environments has resulted in their evolution of photosynthetic and metabolic machinery optimized to different nutrient and light regimes (Lavaud et al., 2004; Gao et al., 2018; Smith et al., 2019; Fisher et al., 2020). In contrast, dinoflagellates are favored following seasonal stratification and nutrient depletion in eutrophic coastal seas, fuel microbial food webs and have the potential to form harmful algal blooms (Margalef, 1978; Smayda, 2002; Sommer et al., 2002; Delwiche, 2007). Diatoms and dinoflagellates have distinct biochemical composition. For example, diatoms contain high levels of 16:1n-7, EPA (eicosapentaenoic acid, 20:5n-3) and brassicasterol/epi-brassicasterol, and high ratios of 16:1n-7/16:0 and EPA/DHA (docosahexaenoic acid, 22:6n-3), while characteristic FAs and sterols of dinoflagellates are 18:0; 18:1, DHA, and dinosterol (Dalsgaard et al., 2003; Volkman, 2003; Bi et al., 2014; Jiang et al., 2016). Such differences in biochemical composition have shown strong effects on trophic carbon transfer between phytoplankton and consumers (Müller-Navarra et al., 2000; Martin-Creuzburg and von Elert, 2009; Malzahn et al., 2010; Bi and Sommer, 2020). Also, on a long-term scale the shift in the diatom-dinoflagellate community have been reported in many areas of the ocean, which were driven by environmental changes induced by anthropogenic and climatic forcing (Schubert et al., 1998; Xing et al., 2016; Yuan et al., 2020).

Temperature and nutrients (N and P) are the most important factors controlling the diatom-dinoflagellate community (Glibert et al., 2011; Cross et al., 2015; Sunagawa et al., 2015; Cloern et al., 2016). The ocean is simulated to be warmed 5- to 7-fold from today to 2100 compared with the change from 1970 until today, according to the RCP8.5 scenario of the high emissions (Bindoff et al., 2019). The external supply of N and P to the ocean has increased, with a projected total input of 50:1 in N:P molar ratios to the ocean by 2050 (Moore et al., 2013). N:P ratios have strong variations, e.g., a molar ratio of 6:1 in the Southeast Pacific Gyre (Bonnet et al., 2008) and up to 100:1 in summer in certain areas of the East China Sea (ECS) (Wu et al., 2016). Warming, eutrophication and imbalanced N:P ratios have caused dramatic changes in the diatom-dinoflagellate community, which is typified by an increase in relative abundance of dinoflagellates compared to diatoms, with dinoflagellates becoming dominant together with diatoms or being the only dominant group (Xie et al., 2015; Liu et al., 2016; Siemering et al., 2016; Carreto et al., 2018).

However, contrasting observations have been obtained on the responses of diatom-dinoflagellate communities to temperature and nutrient availability changes. An increase in temperature generally enhances the abundance of dinoflagellates but not diatoms (Morse et al., 2013; Jiang et al., 2015; Xie et al., 2015); in contrast, an increase in the relative abundance of diatoms vs. dinoflagellates with increasing temperature has been found in different sea areas, such as the northeast Atlantic and North Sea (Hinder et al., 2012) and Chilean Patagonian fjord (Montero et al., 2017). While diatoms usually dominate at high nutrient conditions at both global and local scales (Aubry et al., 2004; Guo et al., 2014a; Rousseaux and Gregg, 2015; Mutshinda et al., 2016), some have reported the dominance of dinoflagellates at high nutrient conditions (Lotze and Milewski, 2004; Liu et al., 2016; Klais et al., 2017). An increasing N:P ratios often negatively correlates with the diatoms/dinoflagellates ratio (Zhang, 2009; Glibert et al., 2011; Guo et al., 2014b); however, a positive correlation has been also found in some coastal areas (Hodgkiss and Ho, 1997; Heisler et al., 2008; Xiao et al., 2013). Such seemingly contradictory findings on diatom-dinoflagellate competition can be largely attributed to the interplay between multiple environmental factors, especially between warming and eutrophication (Lewandowska et al., 2014; Cross et al., 2015; Xiao et al., 2018; Gerhard et al., 2019). While increasing attention has been devoted to experimentally study the effects of temperature and/or nutrient conditions on the diatom-dinoflagellate community (Wang et al., 2006; Lassen et al., 2010; Huang et al., 2012; Bach et al., 2020), our quantitative knowledge of these effects is still inadequate.

**FIGURE 1:**
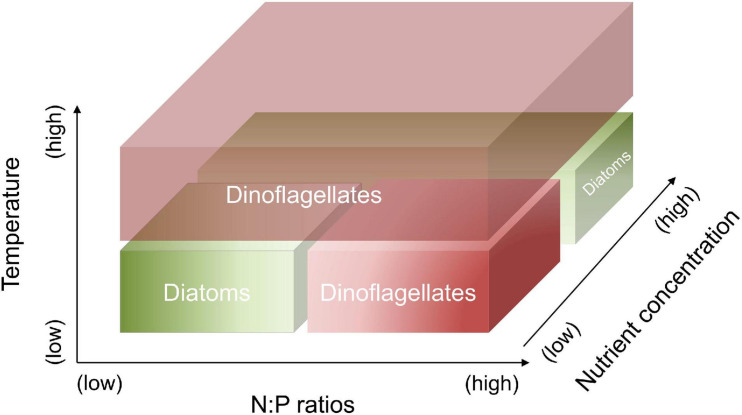
Hypothetic responses of diatoms vs. dinoflagellates to the changes in temperature, N:P supply ratios and nutrient concentrations. Dinoflagellates have the competitive superiority at high temperature, as well as at combined conditions of low temperature, high N:P supply ratios and a low nutrient regime. Diatoms have the competitive superiority at combined conditions of low temperature, low N:P supply ratios and a low nutrient regime, as well as at low temperature and a high nutrient regime.

In this study, laboratory experiments were conducted to investigate the responses of a diatom-dinoflagellate community to the interactions between temperature, N and P concentrations and their ratios. In particular, we focus on quantitatively assessing the changes in elemental stoichiometry and lipid biomarkers associated with the shifts of community composition. We hypothesize that (1) dinoflagellates have the competitive superiority at high temperature, as well as at combined conditions of low temperature, high N:P supply ratios and a low nutrient regime; diatoms have the competitive superiority at combined conditions of low temperature, low N:P supply ratios and a low nutrient regime, as well as at low temperature and a high nutrient regime (Figure 1); and (2) the changes in elemental and biomarker composition can quantify the shifts in a diatom-dinoflagellate community under different temperature and nutrient conditions.

## Materials and Methods

### Experimental Set-Up

The phytoplankton species used in the experiments were the diatom *Phaeodactylum tricornutum* (Code No.: MACC/B254, supplied by the Microalgae Culture Center at the Ocean University of China) and the dinoflagellate *Prorocentrum minimum* (Code No.: HYESL63, isolated from the ECS and supplied by Microalgae Germplasm Bank in the Second Institute of Oceanography, the Ministry of Natural Resources). Both monocultures and bicultures of the two species were exposed to a salinity of 37 psu and a light intensity of 100 μmol photons m^–2^ s^–1^ with a light:dark cycle of 12:12 h in temperature-controlled cabinets of 12, 18, and 24°C. The light intensity used in this study did not limit the growth of *P. tricornutum* and *P. minimum* (Beardall and Morris, 1976; Grzebyk and Berland, 1996). The chosen temperatures cover the optimal values for the growth of the two species (Grzebyk and Berland, 1996; Bojko et al., 2013), and the range of 6°C was set based on the ocean general circulation model under the scenarios of IPCC SRES A1F1 (Lewandowska et al., 2014). In bicultures, two species were mixed from the beginning on in the experiments. The culture medium was prepared according to the modified Provasoli’s medium (Provasoli, 1963; Ismar et al., 2008). Enrichment nutrient solutions were added to sterile filtered (0.2 μm pore size, Sartobran^®^ P 300) sea water. Sodium nitrate and potassium dihydrogen phosphate were added to achieve the molar ratios of 10:1, 24:1, and 63:1 at three different nutrient concentration levels (Table 1). Sodium silicate was added to the medium at a concentration of 880 μmol L^–1^ in all treatments. At the normal nutrient level, N and P concentrations were comparable with those in the f/2 medium, which has been widely used as a general enriched seawater medium for culturing coastal marine algae (Harrison and Berges, 2005), while a 5-fold increase in N and P concentrations from the normal to high nutrient level covered the ranges of nutrient concentration changes in natural conditions, such as in the ECS (Xiao et al., 2018). The intermediate level of N:P supply ratios (24:1) was also consistent with that in the f/2 medium. The culture volume was 200 mL, and each treatment was replicated three times. All culture flasks were carefully agitated twice per day at a set time to minimize sedimentation. Prior to the experiments, the two species were maintained under the tested conditions for more than 12 generations to ensure that lipid composition of phytoplankton reflected acclimated responses (Burkhardt et al., 1999; Reinfelder, 2012).

**TABLE 1:** N and P concentrations (μmol L^–1^) and their molar ratios in bi-algal competition experiments.

Nutrient concentration level		N:P		
		10:1	24:1	63:1
Low	N	35.2	88	88
	P	3.6	3.6	1.4
Normal	N	352	880	880
	P	36	36	14
High	N	1,760	4,400	4,400
	P	180	180	70

Batch cultures were first set for each treatment across a fully factorial combination of three temperatures (12, 18, and 24°C), three N:P supply ratios (molar ratios 10:1, 24:1, and 63:1) and three nutrient concentration levels. The observed maximal growth rate (*μ*_max_, d^–1^) was calculated based on cell numbers within the exponential growth phase of batch cultures (Supplementary Table 1). Once the early stationary phase was reached, semi-continuous cultures were initiated with the algae from batch cultures. For bicultures, the growth of the diatom *P. tricornutum* was used to determine the start of semi-continuous cultures. In semi-continuous cultures, the gross growth rate (*μ*, d^–1^) was set as 20% of *μ*_max_. The volume of the daily renewal medium was determined by multiplying the daily renewal rate (*D*, d^–1^; *D* = 1 – e^–^*^μ × *t*^*, where *t* is renewal interval which was 1 d in the experiments) by the culture volume (200 mL). As both species had different *μ*_max_ in bicultures, *μ* of *P. tricornutum* was used to define the daily renewal rate. Steady state of semi-continuous cultures was assessed based on the net growth rate (*r*, d^–1^), i.e., the difference between *μ* and the loss rate (*r* = *μ* - *D*). In semi-continuous cultures, population losses result from the process of daily renewal incubation water, and the loss rate equals the renewal rate (*D*). At steady state in monocultures, *μ* was equivalent to *D*, and *r* was zero, while in bicultures, a zero net growth rate existed for both species if they coexisted, and for the dominant species when the other was driven to low abundances or excluded.

### Sample Analysis

Algal cells were harvested at steady state in semi-continuous cultures. The following parameters were analyzed: Cell density, POC, PON, POP, FAs, and sterols. Cell density was measured daily during the experiments using an improved Neubauer hemacytometer (Glaswarenfabrik Karl Hecht GmbH) under a microscope (Olympus CX41) (Supplementary Figure 1). Algal cells for elemental and lipid biomarker analysis were harvested by filtration on pre-combusted and hydrochloric acid-treated GF/F filters (Whatman) at the same time. Filtration volumes were 15–30 mL depending on cell density. After filtration, all samples were frozen at -80°C until analysis.

The determination of POC and PON was carried out after Sharp (1974) by gas chromatography in an organic elemental analyzer (Thermo Flash 2000), with organic analytical standards atropine (Thermo Fisher Scientific) and a low organic content soil (Elemental Microanalysis Ltd.) as standards. POP was analyzed by converting organic phosphorus compounds to orthophosphate (Hansen and Koroleff, 1999) and measured colorimetrically at 880 nm with a spectrophotometer (HITACHI U-2910).

Lipid biomarkers (sterols and FAs) were measured according to Zhao et al. (2006). Briefly, freeze-dried samples were extracted with the mixture of dichloromethane and MeOH (3:1, vol/vol) for eight times, with C_19_ *n*-alkanol and nonadecanoic acid (19:0) as internal standards. After hydrolysis with 6% KOH in MeOH, the extracts were separated into polar and apolar fractions using silica gel chromatography. The polar lipid fraction (containing brassicasterol/epi-brassicasterol and dinosterol) was eluted with 22-mL dichloromethane/methanol (95:5, vol/vol) and dried under a gentle N_2_ stream. After elution, the polar fractions were silylated with 80 μL BSTFA (*N, O*-bis(trimethylsilyl)-trifluoroacetamide) at 70°C for 1 h. FAs in the acid fraction were derivatized to corresponding fatty acid methyl esters (FAMEs) with MeOH/HCl (95:5, 12 h at 70°C). Sterols and FAMEs were quantified based on internal standards on a gas chromatograph with flame ionization detection (GC-FID; Agilent Technologies 7890A). The GC columns for sterol and FA analysis were HP-1 (50 m × 0.32 mm i.d., 0.17-μm film thickness; Agilent J&W) and SP-2560 (100 m × 0.25 mm i.d., 0.20-μm film thickness; Supelco), respectively. Sterols were identified according lab standards, while FAs were identified with reference to commercially available standards, Supelco 37 component FAME mixture and Supelco Menhaden fish oil.

### Data Analysis and Statistics

Species interactions were estimated by fitting Lotka-Volterra model (Eqs. 1 and 2):

(1) $$\frac{d_{N_{diatom}}}{d_{t}}\frac{1}{N_{diatom}}=r_{diatom}\frac{K_{diatom}-N_{diatom}-\alpha N_{dino}}{K_{diatom}}$$

(2) $$\frac{d_{N_{dino}}}{d_{t}}\frac{1}{N_{dino}}=r_{dino}\frac{K_{dino}-N_{dino}-\beta N_{diatom}}{K_{dino}}$$

where *N_*dia*__*to*__*m*_* and *N*_*dino*_ (10^4^ cells ml^–1^) represent cell density of the diatom *P. tricornutum* and the dinoflagellate *P. minimum*, respectively, during semi-continuous cultures. $\frac{d_{N_{diatom}}}{d_{t}}$ and $\frac{d_{N_{dino}}}{d_{t}}$ are thus calculated for the period of semi-continuous cultures. *r_*dia*__*to*__*m*_* (d^–1^) and *K_*dia*__*to*__*m*_* (10^4^ cells ml^–1^) are the maximum growth rate and carrying capacity for *P. tricornutum* in monoculture, and *r*_*dino*_ and *K*_*dino*_ for *P. minimum* (Supplementary Table 1). In this model, *α* and *β* are interaction coefficients, with *α* representing the impact of the dinoflagellates on the diatoms and *β* indicating that of the diatoms on the dinoflagellates. In this study, we focused on the impact of the dinoflagellates and the diatoms, as ocean-related global change generally enhances the competitive superiority of dinoflagellates over diatoms in many areas of the ocean (Morse et al., 2013; Xie et al., 2015; Carreto et al., 2018). Thus, we analyzed the responses of *α*. The dinoflagellates have the competitive superiority at *α* > 0; the diatoms have the competitive superiority at *α* < 0; and there is no competitive superiority for either species at *α* = 0.

Generalized linear mixed models (GLMMs) were used to investigate the factors determining phytoplankton competition, stoichiometric and lipid biomarker contents. Response variables were cell density, the interaction coefficient *α*, C:N:P stoichiometric molar ratios, FA biomarkers (16:1/16:0 and EPA/DHA), sterol biomarkers [the ratios of brassicasterol/epi-brassicasterol to (brassicasterol/epi-brassicasterol + dinosterol), B/(B + D)], with temperature, N:P supply ratios and nitrate concentrations as fixed effects. Target distributions were tested and link functions were consequently chosen. For all response variables, models containing first-order effects of the three factors, and second- and third-order interactions of all factors were tested. Model selection with the Akaike Information Criterion corrected (AICc) was used to determine the model that best predicted targets. A lower AICc value represents a better fit of the model, with 10 units or more difference being considered as a reasonable improvement in the models. If the difference in AICc values was smaller than 10 units, the simpler model was chosen, unless significant second- or third-order interactions were detected. Models containing only first-order effects of temperature, N:P supply ratios and nutrient concentrations were selected as the best models for interaction coefficient *α*, POC/PON, PON/POP, 16:1/16:0, and EPA/DHA, while that containing first-order effects and second-order interactions was chosen for B/(B + D) (Supplementary Table 2).

Generalized additive models (GAMs) were used to determine the amplitude of the response of the competition coefficient *α* to nitrate concentration under different temperatures, i.e., the difference between *α* at the strongest and the weakest effect of nitrate concentration under certain temperature condition. The GAMs were fitted separately for each of the three temperatures, as well as to the data from all temperature treatments. The partial additive effect of nitrate concentration on the coefficient *α* was visualized by GAM curves of partial residuals of the non-linear terms. The GAM results show that the individual temperature models explained 34.3–73.5% of the deviance in *α*, while the joined model with three temperatures explained 33.5% of the deviance (Table 2). Also, *r*^2^ and generalized cross-validation (GCV)-values were better in the individual temperature models than the joined model in most cases. Thus, the individual temperature models were selected, from which GAM curves of partial residuals of the non-linear estimation are presented in our study.

**TABLE 2:** Generalized additive model (GAM) results.

Treatment	Intercept	Nitrate concentration		GCV	*r*^2^ (adj.)	Deviance explained (%)
	Estimate ± SE	edf	*p*-value			
12°C	7.36 ± 11.64	3.391	<0.001	3725.9	0.684	73.5
18°C	–24.412 ± 8.522	3.883	0.003	2324.7	0.434	52.2
24°C	0.582 ± 5.530	2.036	0.030	840.22	0.279	34.3
Joined model (three temperatures)	–6.373 ± 6.618	3.769	<0.001	3376.7	0.298	33.5

*Intercept (estimate ± standard error), estimated degrees of freedom (edf), and significance (*p*-value) of nitrate concentration for the competition coefficient *α* under different temperatures. GCV, generalized cross-validation.*

Linear regressions were conducted to test the relationship between PON/POP (and 16:1/16:0 and EPA/DHA) and the cell ratios of diatoms to dinoflagellates. Also, the correlations between B/(B + D) and the cell ratios of diatoms to the sum of diatoms and dinoflagellates were analyzed.

GLMMs and linear regressions were conducted in SPSS 19.0 (IBM Corporation). GAM was conducted using the “gam” function from the package mgcv (Wood, 2017) in R version 3.5.1 (R Development Core Team, 2010). Significance level was set to *p* < 0.05 in all statistical tests.

## Results

### Diatom-Dinoflagellate Competition Response

**FIGURE 2:**
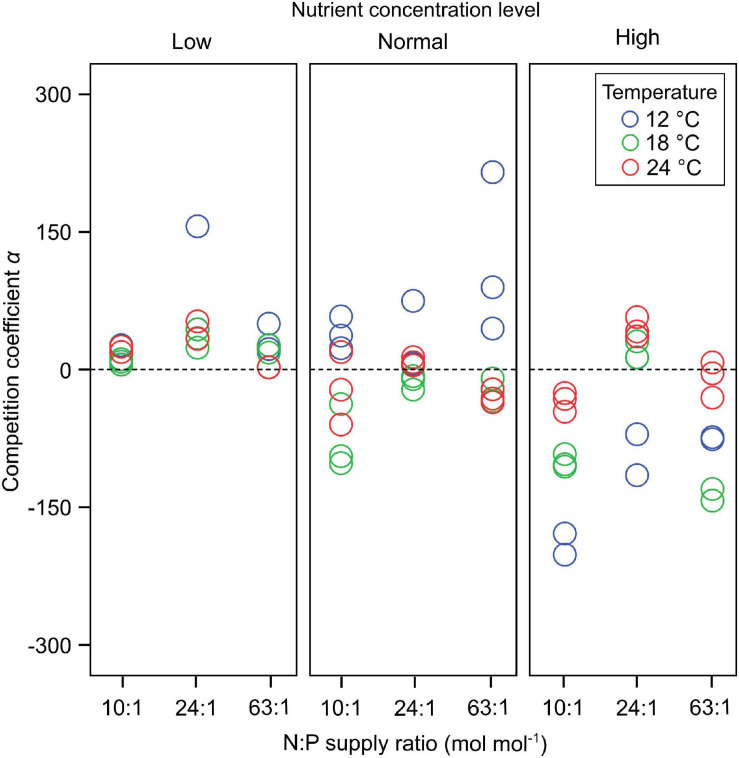
The responses of the interaction coefficient *α* to temperature, N:P supply ratios and nutrient concentrations in the bicultures of *Phaeodactylum tricornutum* and *Prorocentrum minimum*. At *α* > 0 dinoflagellates have the competitive superiority, while diatoms have the competitive superiority at *α* < 0. There is no competitive superiority for either species at *α* = 0.

Dinoflagellates had the competitive superiority (*α* > 0) under low nutrient concentrations, but diatoms had the competitive superiority (*α* < 0) under high nutrient concentrations (Figure 2). The interaction coefficient α responded significantly to nutrient concentration changes (GLMMs; Table 3), showing a decrease as nutrient concentrations increased (Figure 2), while its responses to temperature and N:P supply ratios were not significant.

**TABLE 3:** Results of the selected GLMMs testing for the effects of temperature, N:P supply ratios, and nitrate concentrations on interaction coefficient *α*, POC/PON, PON/POP, 16:1/16:0, EPA/DHA and the ratios of brassicasterol/epi-brassicasterol to (brassicasterol/epi-brassicasterol + dinosterol) [B/(B + D)] in bicultures.

Variable	Factor	Coefficient	*SE*	*t*	*p*	*n*
*α*	Intercept	2.662	31.767	0.084	0.933	72
	T	–0.343	1.590	–0.216	0.830	
	Nut. Conc.	–0.013	0.005	–2.726	**0.008**	
	N:P	0.494	0.348	1.421	0.160	
POC/PON	Intercept	1.112	0.061	18.168	<0.001	79
	T	<0.001	0.003	–0.078	0.938	
	Nut. Conc.	<0.001	<0.001	–7.976	**<0.001**	
	N:P	–0.001	0.001	–1.521	0.132	
PON/POP	Intercept	1.248	0.138	9.062	<0.001	64
	T	0.002	0.007	0.224	0.823	
	Nut. Conc.	<0.001	<0.001	–5.890	**<0.001**	
	N:P	0.008	0.002	4.724	**<0.001**	
16:1/16:0	Intercept	0.335	0.120	2.783	0.007	62
	T	–0.010	0.006	–1.656	0.103	
	Nut. Conc.	<0.001	<0.001	4.199	**<0.001**	
	N:P	–0.002	0.001	–1.879	0.065	
EPA/DHA	Intercept	5.231	2.298	2.276	0.027	62
	T	0.233	0.113	2.071	**0.043**	
	Nut. Conc.	0.003	<0.001	7.445	**<0.001**	
	N:P	–0.070	0.025	–2.767	**0.008**	
B/(B + D)	Intercept	0.071	0.078	0.908	0.367	79
	T	–0.007	0.004	–1.640	0.105	
	Nut. Conc.	<0.001	<0.001	–1.115	0.268	
	N:P	<0.001	0.002	–0.008	0.993	
	T × Nut. Conc.	<0.001	<0.001	2.090	**0.040**	
	T × N:P	<0.001	<0.001	–1.164	0.248	
	Nut. Conc. × N:P	<0.001	<0.001	1.191	0.238	

*Significant *p*-values are shown in bold. T, temperature; N:P, N:P supply ratios; Nut. Conc., nitrate concentration.*

**FIGURE 3:**
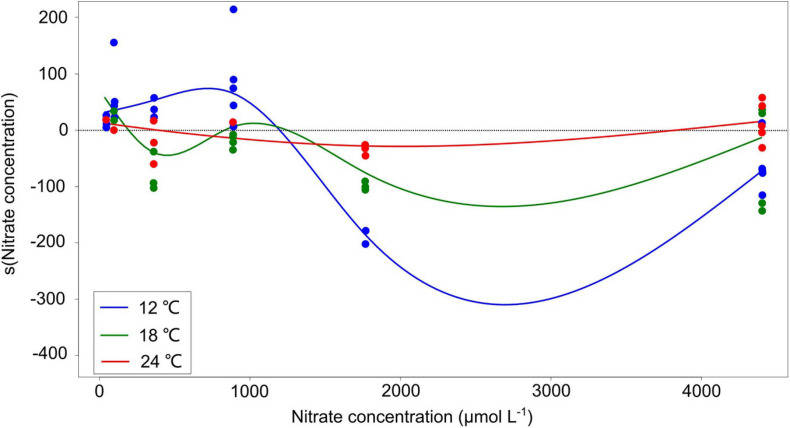
Partial residuals of the non-linear estimation from the generalized additive models (GAMs) with smoothing splines. The y-axis indicates the partial additive effect of nitrate concentration on interaction coefficient *α*, and s () is the shorthand for fitting smoothing splines. The solid curves are the estimates of the smooth with GAMs, and the dots represent observed data points. The summary of GAM results is shown in Table 2.

The response amplitude of the competition coefficient *α* to nitrate concentration decreased with increasing temperature (Figure 3). The y-axis of Figure 3 indicates the partial additive effect of nitrate concentration on interaction coefficient α, with s () as the shorthand for fitting smoothing splines. While temperature *per se* had no overall effect on coefficient response, it was the amplitude of the response that was affected, not the direct response. Indeed, we observed that the interaction coefficient *α* also varied with temperature, showing higher values at the lowest temperature under normal-nutrient conditions, but higher values at higher temperatures under the highest nutrient concentrations (*α* > 0 indicating the competitive superiority of dinoflagellates; Figure 2).

### C:N:P Stoichiometry of Particulate Organic Matter

**FIGURE 4:**
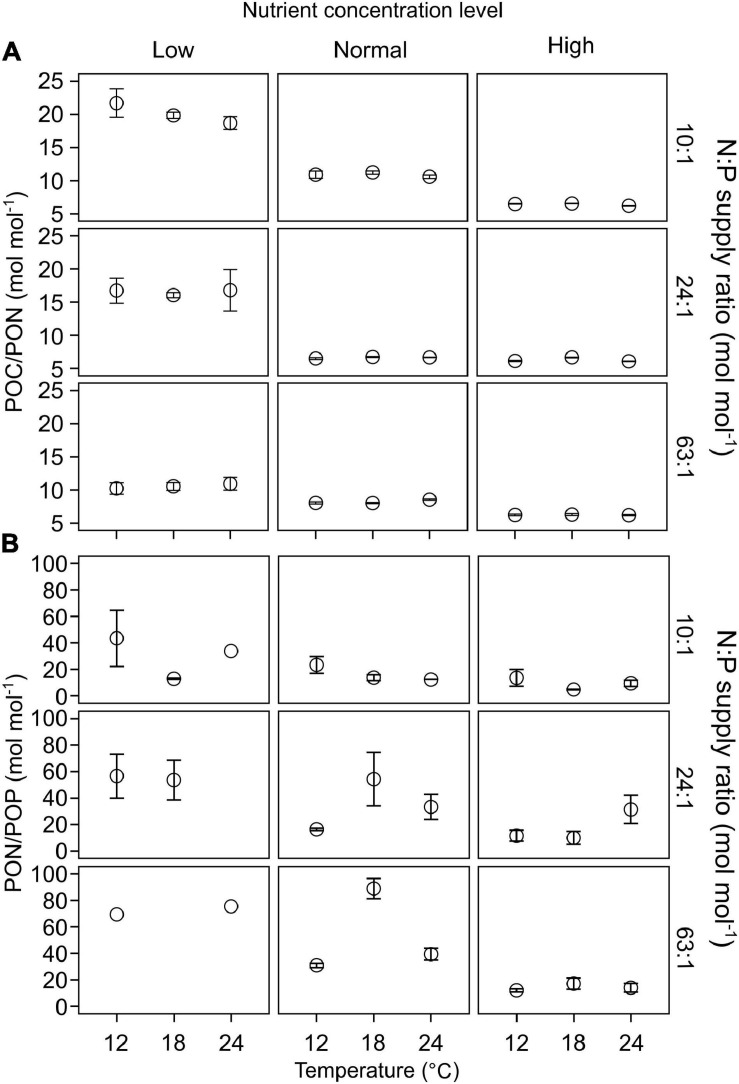
The responses of C:N:P stoichiometry in particulate organic matters (mean ± SE) to temperature, N:P supply ratios and nutrient concentrations in the bicultures of *Phaeodactylum tricornutum* and *Prorocentrum minimum*: **(A)** POC/PON and **(B)** PON/POP.

POC/PON responded significantly to changes in nutrient concentrations, but not to temperature or N:P supply ratios (GLMMs; Table 3 and Supplementary Table 1). POC/PON decreased with increasing nutrient concentrations (Figure 4A).

**FIGURE 5:**
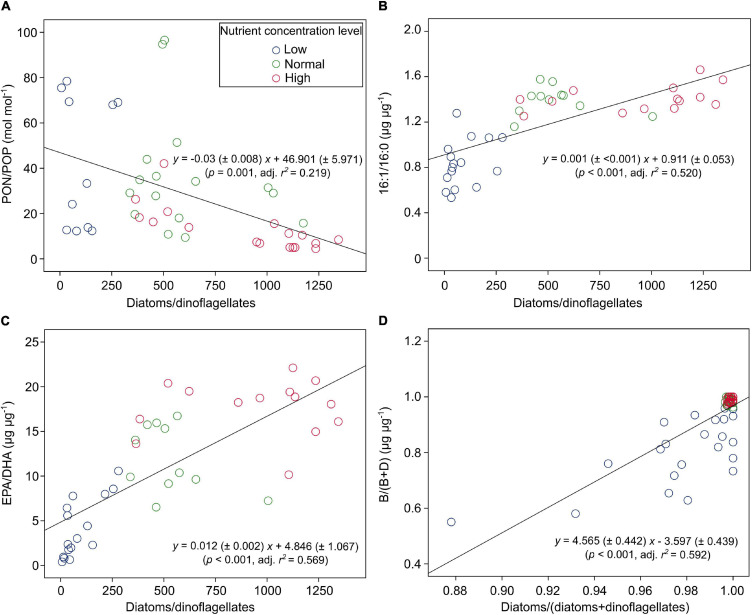
The correlations between PON/POP (and lipid biomarker ratios) and cell density ratios of diatoms/dinoflagellates [or diatoms/(diatoms + dinoflagellates)] under three levels of nutrient concentrations over the entire ranges of temperature and N:P supply ratios in the bicultures of *Phaeodactylum tricornutum* and *Prorocentrum minimum*: **(A)** PON/POP, **(B)** 16:1/16:0, **(C)** EPA/DHA, and **(D)** the ratios of brassicasterol/epi-brassicasterol to (brassicasterol/epi-brassicasterol + dinosterol) [B/(B + D)].

PON/POP responded significantly to nutrient concentrations and N:P supply ratios, but not to temperature (GLMMs; Table 3), showing general higher values at lower nutrient concentrations and higher N:P supply ratios (Figure 4B and Supplementary Figure 2). PON/POP showed a significant negative correlation with increasing diatoms/dinoflagellates cell ratios (Linear regression, *p* = 0.001; Figure 5A).

### Lipid Biomarkers—Fatty Acids and Sterols

**FIGURE 6:**
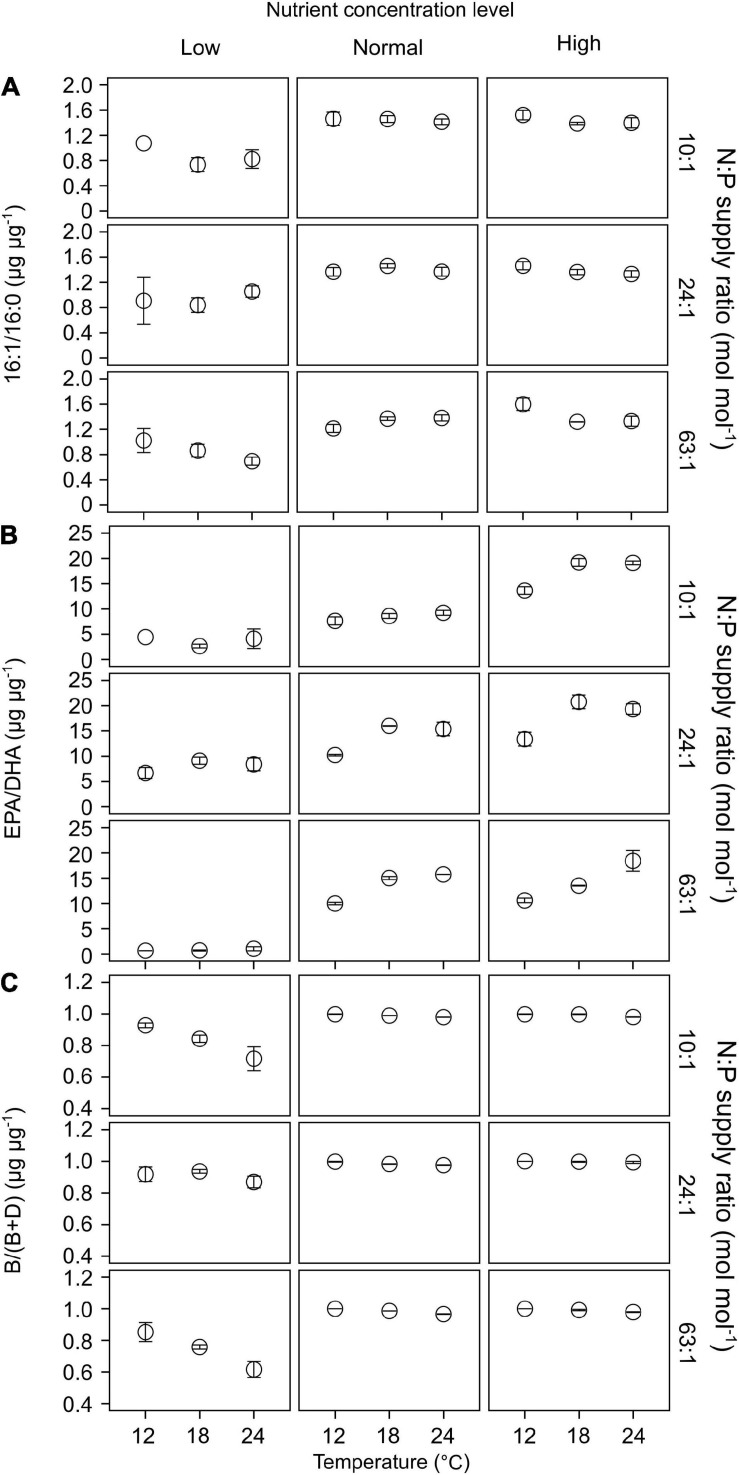
The responses of lipid biomarker ratios (mean ± SE) to temperature, N:P supply ratios and nutrient concentrations in the bicultures of *Phaeodactylum tricornutum* and *Prorocentrum minimum*: **(A)** 16:1/16:0, **(B)** EPA/DHA, and **(C)** the ratios of brassicasterol/epi-brassicasterol to (brassicasterol/epi-brassicasterol + dinosterol) [B/(B + D)].

Of the 22 FA components identified in our study, seven contributed largely to TFAs (>5% of TFAs) over the entire ranges of temperature, N:P supply ratios and nutrient concentration levels (Supplementary Table 3). These included the saturated 14:0 and 16:0, monounsaturated 16:1n-7 and 18:1n-9c, and polyunsaturated 18:2n-6c, EPA and DHA. The ratios of both 16:1n-7 to 16:0 (16:1/16:0) and EPA to DHA (EPA/DHA) responded significantly to the changes in nutrient concentrations (GLMMs; Table 3), showing an increase with increasing nutrient concentrations (Figures 6A,B). Moreover, EPA/DHA also changed significantly with temperature and N:P supply ratios, showing overall higher values at higher temperatures and lower N:P ratios. 16:1/16:0 and EPA/DHA correlated positively with the cell density ratios of diatoms/dinoflagellates (Linear regression, *p* < 0.001; Figures 5B,C).

Brassicasterol and dinosterol were two major sterols detected in bicultures (Supplementary Table 3). The ratios of brassicasterol/epi-brassicasterol to (brassicasterol/epi-brassicasterol + dinosterol) [B/(B + D)] responded significantly to the interactions of temperature and nutrient concentrations (GLMMs; Table 3), showing a decrease with increasing temperature at the lowest nutrient concentration (Figure 6C). B/(B + D) showed significant positive correlations with the cell density ratios of diatoms to the sum of diatoms and dinoflagellates (Linear regression, *p* < 0.001; Figure 5D).

## Discussion

This study shows that the effect of nutrient concentrations is key in regulating diatom-dinoflagellate competition, as revealed by the competition coefficient, elemental stoichiometry and lipid biomarkers (FAs and sterols). Significant effects of temperature and N:P supply ratios were only observed on certain competition indices. Specifically, our results show competitive superiority of the diatom *P. tricornutum* over the dinoflagellate *P. minimum* at high nutrient concentrations, which holds independently of temperature conditions and N:P supply ratios in the culture medium. Although temperature showed non-significant effects on the competition coefficient, the variation in temperature altered the response amplitude of diatom-dinoflagellate competition to nutrient concentration changes, showing the interaction between the two environmental factors, consistent with previous findings in eutrophic coastal areas (Xiao et al., 2018). The changes in temperature and N:P supply ratios had significant effects on elemental and lipid composition in the diatom-dinoflagellate community, e.g., the increase in N:P ratios causing significant higher PON/POP at the two low nutrient levels. Such variations in chemical composition of the diatom-dinoflagellate community significantly correlated with the changes in the relative abundance of diatoms and dinoflagellates.

### Diatom-Dinoflagellate Competition Under Different Temperature and Nutrient Regimes

Nutrient concentrations had significant effects on the interaction coefficient *α* of the diatom-dinoflagellate competition (Table 3). Specifically, the responses of the interaction coefficient *α* to nitrate concentration reveal two scenarios. On the one hand, interaction coefficient α showed a switch from a competitive superiority of the dinoflagellates to the diatoms as nitrate concentration increased at the low nutrient regime (Figure 3), indicating that the dinoflagellates favor low nutrient condition, but the diatoms favor high nutrients. Our results are consistent with the succession trends in phytoplankton communities in the world ocean where high concentrations of nitrate are encountered, mainly in highly eutrophic bays and river inlets (Aubry et al., 2004; Gettings et al., 2014; Mutshinda et al., 2016; Barcelos e Ramos et al., 2017; Ratmaya et al., 2019). For example, the Bay of Brest received high-nitrate-loaded freshwater (>700 μmol L^–1^) in winter in 1990s, which supported a diatom-dominated spring bloom (Del Amo et al., 1997). Comparable situations have been reported for the Changjiang Estuary and adjacent shelf of the ECS and southern Yellow Sea, where diatoms dominated within river plume and high-DIN regions (up to 50 μmol L^–1^), while the density of dinoflagellates was typically high at low-nutrient areas outsides diatom-blooming zones (Jiang et al., 2015, 2019). The diatom-dinoflagellate succession along nitrate concentration changes aligns with ecological traits defined by Margalef (1978). The diatom *P. tricornutum* appeared to have high requirements for nutrients (*r*-strategy), and the dinoflagellate *P. minimum* outcompeted the diatoms at low nutrient conditions (*K*-strategy). Genetic analysis has shown the remodeling of intermediate metabolism in *P. tricornutum*, allowing the diatoms to efficiently respond to changes in nitrogen availability and thereby to become ecologically successful in the aquatic environments where nitrogen can be introduced rapidly through turbulence (Levitan et al., 2015; Smith et al., 2019). In contrast, dinoflagellate abundance has shown a positive correlation with N-acquisition genes, which is linked to the competitive advantage of dinoflagellates at low nutrient conditions (Zhuang et al., 2015; Elferink et al., 2020).

On the other hand, the response amplitude of the interaction coefficient *α* to nitrate concentration shows a switch back toward competitive superiority of the dinoflagellates at the high nitrate regime (Figure 3). Note that only at the highest temperature (24°C) and only at the very high nitrate concentrations, the partial residuals of the non-linear terms in the GAM plot truly became positive again (the red curve in Figure 3). The results are in agreement with field observations, which show that climate warming and high nutrient concentration play a dominant role in the phytoplankton assemblage changing from the diatoms to the dinoflagellates in different regions, e.g., the Chesapeake Bay (Malone, 1992), the north Atlantic (Leterme et al., 2005), the western English Channel (Xie et al., 2015), and the Baltic Sea (Spilling et al., 2018). In particular, the extent of the increase in nutrient concentrations in our study (5-fold higher than f/2 medium) is comparable with that during 2002–2015 in the ECS (∼5- to 10-fold increase; NO_2_^–^ + NO_3_^–^: between 0–1.0 and 10–50 μmol L^–1^; PO_4_^3–^: between 0–0.08 and 0.8–1.5 μmol L^–1^) (Xiao et al., 2018). Consistent with our study, model simulations on the diatom-dinoflagellate competition demonstrated that the spring dinoflagellate bloom in the coastal ECS is projected to increase at high temperature (>∼24°C) and high nutrient concentrations (PO_4_^3–^: 0.25 and 0.6 μmol L^–1^, ∼5- to 10-fold more than the lowest concentration 0.05 μmol L^–1^) (Xiao et al., 2018). An explanation for the switch back toward competitive superiority of the dinoflagellates is that the highest temperature 24°C in our study is above the temperature optimum (around 20°C) for the growth of *P. tricornutum* (William and Morris, 1982), while it is within the higher optimal temperatures for *P. minimum* (around 18–26°C) (Grzebyk and Berland, 1996). Furthermore, *P. minimum* can also form toxic blooms, particularly occurred under warm and eutrophic coastal waters in subtropical and temperate climatic zones (Heil et al., 2005). It has been observed that allelochemicals produced by *P. minimum* significantly inhibited the growth of the diatom *Skeletonema costatum* (Tameishi et al., 2009). For another dinoflagellate *Prorocentrum lima*, increasing temperature may lead to higher cellular toxin levels (Aquino-Cruz et al., 2018). Therefore, the allelopathic effects of *P. minimum* on *P. tricornutum* may be partially attributed to the competitive dominance of *P. minimum* at the highest temperature and nitrate concentrations in our study, which should be tested in further studies.

In summary, we observed a switch from a competitive superiority of the dinoflagellates to the diatoms with increasing nutrient concentrations, and the shift back to dinoflagellates at very high nutrient levels and high temperatures. While the former responses in the diatom-dinoflagellate competition have been previously observed, the latter has not been even covered in classical competition theories, which focused on resource competition and were not coupled with increasing temperature (Tilman, 1982; Sommer, 1989; Smayda and Reynolds, 2003). Our results reveal strong impacts of temperature and nutrient concentrations on diatom-dinoflagellate competition, which are consistent with previous studies, indicating that dinoflagellates are more competitive than diatoms and often form harmful algal blooms under high nutrients and warming conditions, particularly in highly eutrophic bays and coastal areas (Xiao et al., 2018; Glibert, 2020).

### Stoichiometric C:N:P and Diatom-Dinoflagellate Competition

We found significant responses of POC/PON and PON/POP to the changes in nutrient concentrations and N:P supply ratios (only for PON/POP), showing lower values at higher nutrient concentrations (Table 3 and Figure 4), but there were no significant responses to temperature changes. In support, a recent meta-analysis demonstrated that the availability of dissolved inorganic phosphorus and nitrogen is the most significant and shared environmental driver of phytoplankton C:N:P, with POP:POC and PON:POC positively related to the increased in nutrient availability (Tanioka and Matsumoto, 2020). These results are consistent with the nutrient supply hypothesis, which predicts the dependence of ecosystem stoichiometric flexibility on dissolved nutrient concentrations (Galbraith and Martiny, 2015; Garcia et al., 2018). Contrary to our findings, the effects of temperature on phytoplankton stoichiometric variation has been also observed (Toseland et al., 2013; Yvon-Durocher et al., 2015; Bi et al., 2017). For example, the optimal N:P (i.e., threshold N:P at which N and P are co-limiting) is temperature dependent in the green alga *Chlamydomonas reinhardtii* (Thrane et al., 2017). Because nutrient availability can change the extent or even reverse the effects of temperature on phytoplankton C:N:P stoichiometry (Bi et al., 2017), the effects of temperature may be more pronounced under certain nutrient conditions. Nevertheless, our results illustrate that nutrient concentrations highly regulate stoichiometric C:N:P in the diatom-dinoflagellate bicultures.

Furthermore, PON/POP correlated negatively with cell density ratios of diatoms/dinoflagellates in our study (Figure 5A). In line with previous studies (e.g., Weber and Deutsch, 2010; Martiny et al., 2013; Martiny et al., 2016), our results indicate the link between elemental stoichiometry and phytoplankton community structures, in this case, reduced PON/POP was associated with an elevated contribution of diatoms to the diatom-dinoflagellate bicultures. This linkage can be explained by phytoplankton group-specific C:N:P stoichiometry, i.e., lower PON/POP in diatoms than other phytoplankton groups (Quigg et al., 2003; Weber and Deutsch, 2010; Sharoni and Halevy, 2020). Differences in stoichiometric C:N:P composition between phytoplankton groups reflect the variations in biochemical composition, such as protein, ribosomal RNA and lipids (Geider and La Roche, 2002; Sterner and Elser, 2002; Loladze and Elser, 2011; Bi et al., 2014), and in luxury consumption of nutrients, e.g., the accumulation of phosphorus storage pools like polyphosphate (Sterner and Elser, 2002; Martin et al., 2014; Lin et al., 2016). It has been found that residual P (intracellular storage or surface adsorption of inorganic P) rather than nucleic acids and phospholipids dominates the variability in phytoplankton PON/POP during N starvation, with more than 80% of cellular P allocated to residual P in diatoms and only 60% in prasinophytes (Liefer et al., 2019). Such mechanistic insights of residual P pools may therefore indicate a greater luxury uptake of P in diatoms than dinoflagellates, especially under high nutrient concentrations in our study, where diatoms became dominant associated with low PON/POP.

Overall, our study shows that stoichiometric C:N:P varied significantly with changes in N and P concentrations, indicating the fundamental roles of nutrient availability in phytoplankton stoichiometry. Also, PON/POP responded positively to increasing N:P supply ratios, which was more pronounced at low levels of N and P (≤the concentrations in the commonly used f/2 medium) (Supplementary Figure 2). The optimal allocation strategy of N and P in cellular machinery may decouple PON/POP from N:P supply ratios at the highest nutrient level in our study (Klausmeier et al., 2004). Moreover, we found that C:N:P stoichiometry significantly correlated with the shift in the diatom-dinoflagellate community, revealing important implications for marine biogeochemistry and food web dynamics. For example, the competitive superiority of diatoms with low PON/POP can reduce the phosphorus available for nitrogen fixation, with the opposite situation for the competitive superiority of dinoflagellates (Mills and Arrigo, 2010). The relatively low POC/PON at the competitive superiority of the diatoms represents a high elemental quality of food for zooplankton, as high N-rich compounds promote the growth and reproduction of consumers; in contrast, the high POC/PON at the competitive superiority of the dinoflagellates reveals an inferior quality of food (Anderson et al., 2004). The low elemental quality of food may reduce the production of higher trophic levels. An example is the long-term change in the copepod communities in the southern German Bight, where the decrease in elemental food quality of phytoplankton has been suggested to cause a strong decrease in calanoid copepod densities and a decline in herring recruitment during 1975–2011 (Boersma et al., 2015).

### Lipid Biomarkers and Diatom-Dinoflagellate Competition

#### FA Biomarkers

In our study, the two FA biomarkers, 16:1/16:0 and EPA/DHA, had highly significant responses to the changes in nutrient concentrations, showing higher values at higher nutrient concentrations (Table 3 and Figures 6A,B). Similarly, the interaction coefficient *α* also responded significantly to nutrient concentrations in our study. Indeed, the two FA biomarkers correlated positively with cell density ratios of diatoms/dinoflagellates (Figures 5B,C). Field and laboratory studies have shown that high values of 16:1/16:0 and EPA/DHA can indicate the dominance of diatoms vs. dinoflagellates in phytoplankton communities (Reuss and Poulsen, 2002; Cañavate, 2019; Kohlbach et al., 2021). Our data add to previous findings, suggesting a strong correlation between FA composition and phytoplankton community structure over large temperature and nutrient ranges (Dalsgaard et al., 2003; Galloway and Winder, 2015).

Furthermore, we found that EPA/DHA responded significantly to temperature and N:P supply ratios (Table 3), being higher at higher temperatures and lower N:P supply ratios under high nutrient regimes (Figure 6B); however, the interaction coefficient α showed non-significant responses to temperature or N:P supply ratios. As the diatoms were overall dominant at higher nutrient concentrations, the observed changes in EPA/DHA preliminarily reflected the responses of diatom FAs to environmental changes. On top of phylogeny, environmental conditions can also determine phytoplankton FA composition (reviwed by Dalsgaard et al., 2003; Galloway and Winder, 2015; Jónasdóttir, 2019). Previous field studies reported that the main driver of FA composition of phytoplankton was environmental changes, such as seasonal fluctuations in freshwater inflow in San Francisco Bay (Canuel, 2001), and nutrient availability in coastal areas of the Western English Channel (White et al., 2015). Trommer et al. (2019) reported that the shifts in PUFA composition in lake Brunnensee in Germany were independent of algal group succession, but was rather dependent on the changes in per-cell PUFA contents in phytoplankton in response to an increase in nitrogen supply. Our laboratory experiments further quantified the relationship between FA markers and cell density ratios of diatoms/dinoflagellates, showing that the relationship between EPA/DHA and diatoms/dinoflagellates was highly discrete at the highest nutrient concentration (Figure 5C), which can be attributed to the significant effects of temperature and N:P supply ratios (Table 3 and Figure 6B). Our results thus suggest that the effects of environmental factors on phytoplankton FA composition can to some extent modify the relationship between FA biomarkers and phytoplankton community structure.

#### Sterol Biomarkers

The ratios of brassicasterol/epi-brassicasterol to (brassicasterol/epi-brassicasterol + dinosterol) [B/(B + D)] responded significantly to the interactions between temperature and nutrient concentrations, showing a clear decrease with increasing temperature at the lowest nutrient concentration (Figure 6C). Such a decrease in B/(B + D) was associated with a strong increase in carbon-normalized dinosterol contents, but no clear change in brassicasterol/epi-brassicasterol contents (Supplementary Figure 3), in agreement with the competitive superiority of dinoflagellates at the lowest nutrient concentration (Figure 2).

We further showed that B/(B + D) had a good correlation with cell density ratios of diatoms to the sum of two species density (Figure 5D). Our laboratory results are consistent with those in field studies, which have shown the applicability of brassicasterol and dinosterol as proxies of community structure of diatoms and dinoflagellates in suspended particles, e.g., in the Crozet plateau, Southern Ocean (Hernandez et al., 2008), the West Pacific (Dong et al., 2012), and the ECS (Wu et al., 2016; Bi et al., 2018). The present study provides strong evidence for the application of brassicasterol and dinosterol to reveal community structures of diatoms and dinoflagellates under highly variable environmental conditions.

To summarize, our study shows that nutrient concentrations had significant effects on all biomarkers of FAs and sterols [16:1/16:0, EPA/DHA and B/(B + D)], while temperature and N:P supply ratios showed a significant influence only on certain biomarker indices. The variations in lipid biomarkers were overall consistent with those in cell density ratios between diatoms to dinoflagellates. Our results reveal strong ecological relevance for the understanding of the roles of lipid biomarkers in food webs. EPA and DHA are particularly important for animals to meet their needs for population growth and reproduction (Arts et al., 2001; Arendt et al., 2005; Jónasdóttir et al., 2009; Malzahn and Boersma, 2012). These two essential FAs play important but different ecological roles in food webs, showing different distribution patterns within food webs, e.g., EPA highly retained in zooplankton but DHA in fish (Kainz et al., 2004). It has been recently observed that flagellate-dominated phytoplankton communities in summer conferred a higher nutritional value, i.e., higher DHA/EPA ratio, compared to diatom-dominated community in spring, indicating a better quality of zooplankton for fish in summer in the Strait of Georgia in northeast Pacific (Costalago et al., 2020). Nevertheless, our results show significant correlations between lipid biomarkers and diatom-dinoflagellate community composition over wide ranges of temperature and nutrient conditions, indicating that the shift in diatom-dinoflagellate community can result in the fluctuations in the dietary lipids and consequently alter the production of higher trophic levels.

## Conclusion

This study demonstrates that diatom-dinoflagellate competition responded strongly to the changes in nutrient concentrations, while the amplitude of this response was modified by temperature. Specifically, our results show a switch from a competitive superiority of dinoflagellates to diatoms with increasing nitrate concentrations at the low nutrient regime, and a switch back toward the competitive superiority of the dinoflagellates at the high nitrate regime and the highest temperature. These results are consistent with previous field observations, suggesting that dinoflagellates are more competitive than diatoms under high nutrients and warming conditions, particularly in highly eutrophic bays and coasts. Our findings present an important step to quantitatively assess the effects of multiple environmental drivers on diatom-dinoflagellate competition.

Furthermore, elemental stoichiometry and lipid biomarkers in bicultures changed significantly with nutrient concentrations, but significant effects of temperature and N:P supply ratios were only observed on certain parameters. We quantified the relationship between cell density ratios of the two species and their chemical composition in bicultures, showing that the shift in the diatom-dinoflagellate community was significantly correlated with the fluctuations in elemental and biochemical composition over wide ranges of temperature, N and P concentrations and N:P ratios studied in this work. The variations in elemental and biochemical composition reveal a potential far-reaching consequence of the diatom-dinoflagellate succession on biogeochemical cycles and ecological function, e.g., influencing the process of nitrogen fixation, and changing nutritional quality of phytoplankton for higher trophic levels. In addition to the importance of species-specific responses in phytoplankton which is not the focus of this study, our results based on two typical species are very consistent with field observations and ecological modelings. Future work should thoroughly focus on other species and more complex phytoplankton communities to refine our understanding of biogeochemical and ecological consequences of the diatom-dinoflagellate competition.

## Data Availability Statement

The raw data supporting the conclusions of this article will be made available by the authors, without undue reservation. Data supporting the conclusions are also publicly available at https://doi.org/10.5281/zenodo.5205293.

## Author Contributions

RB and MZ designed the study. RB and ZC performed the experiments, with assistance of HZ and YD. SI-R and US provided comments on the data analysis. RB wrote the manuscript with contributions from all co-authors. All authors contributed to the article and approved the submitted version.

## Conflict of Interest

The authors declare that the research was conducted in the absence of any commercial or financial relationships that could be construed as a potential conflict of interest.

## Publisher’s Note

All claims expressed in this article are solely those of the authors and do not necessarily represent those of their affiliated organizations, or those of the publisher, the editors and the reviewers. Any product that may be evaluated in this article, or claim that may be made by its manufacturer, is not guaranteed or endorsed by the publisher.
