# Supplementary material for: Responses of Marine Diatom-Dinoflagellate Competition to Multiple Environmental Drivers: Abundance, Elemental, and Biochemical Aspects
Source: Front Microbiol. 2021 Aug 30;12:731786. doi: 10.3389/fmicb.2021.731786 (PMC8435848; doi:10.3389/fmicb.2021.731786)
Supplement: Supplementary file 1 [file Data_Sheet_1.docx]

Supplementary Material

Supplementary Figure 1


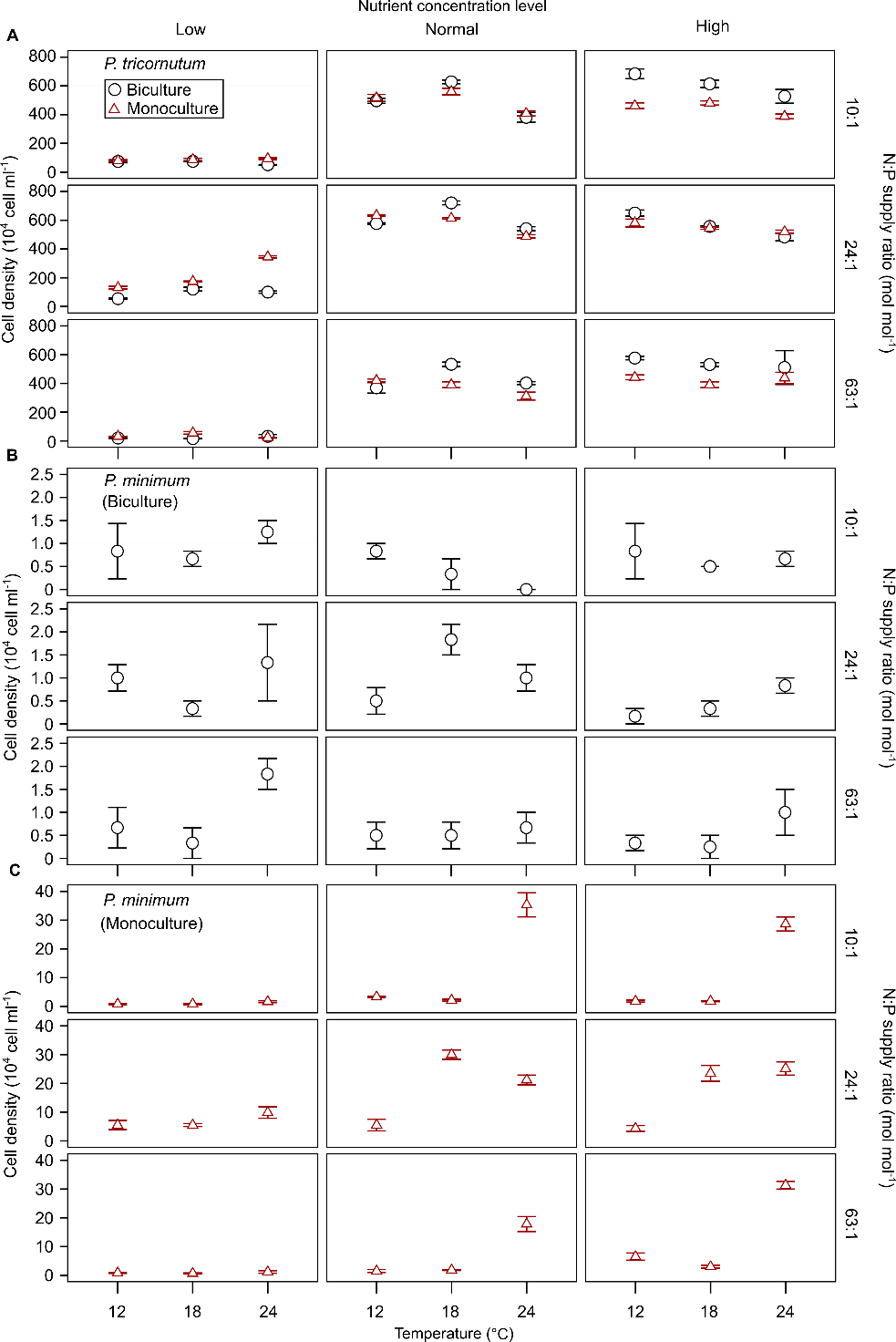


**Supplementary Figure 1.** Cell density of *Phaeodactylum tricornutum* (A) and *Prorocentrum minimum* (B and C) at steady state in the different treatments in bicultures and mono-cultures.

**Supplementary Figure 2**


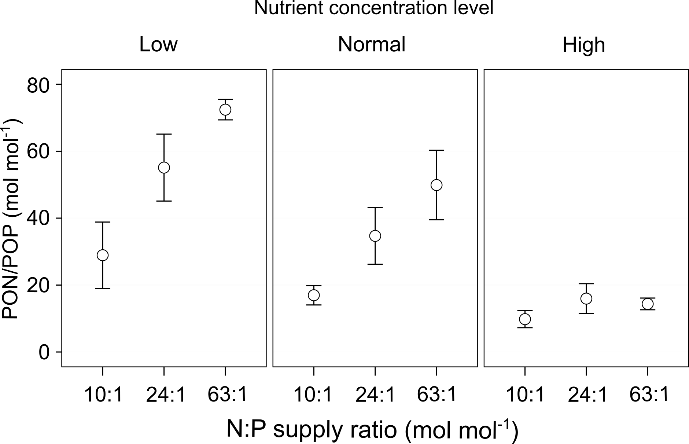


**Supplementary Figure 2.** The responses of PON/POP (mean ± SE) to N:P supply ratios under different nutrient concentration levels across three temperature levels in bicultures.

**Supplementary Figure 3**


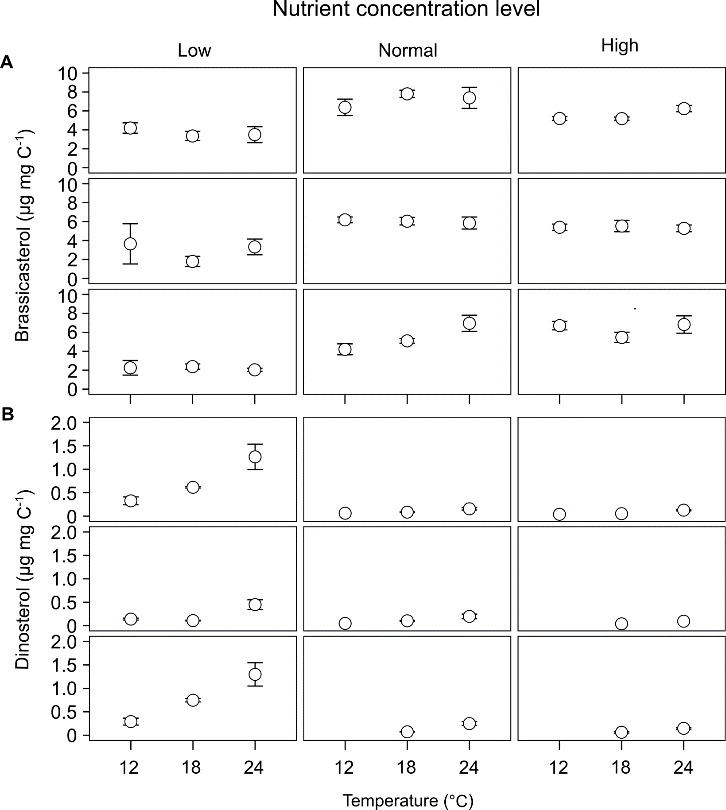


**Supplementary Figure 3.** The responses of carbon-normalized contents of brassicasterol/epi-brassicasterol and dinosterol (mean ± SE) to temperature, N:P supply ratios and nutrient concentrations in the bicultures of *Phaeodactylum tricornutum* and *Prorocentrum minimum*.

**Supplementary Table 1.** The observed maximal growth rate (*μ*_max_, d^-1^) of *Phaeodactylum tricornutum* in batch cultures, and the gross growth rate (*μ*, d^-1^), the daily renewal rate (*D*, d^-1^), the cell density of the diatom *P. tricornutum* and the dinoflagellate *Prorocentrum minimum* at day 0 (*N_diatom0_* and *N_dino0_*) and steady state (*N_diatom1_* and *N_dino1_*; 10^4^ cells ml^-1^) during semi-continuous cultures under different temperatures (12, 18 and 24 ^o^C), N:P supply ratios (molar ratios 10:1, 24:1 and 63:1) and nutrient concentrations (low, normal and high levels) in bicultures of *P. tricornutum* and *P. minimum*. *r_diatom_* (d^-1^) and *K_diatom_* (10^4^ cells ml^-1^) are the maximum growth rate and carrying capacity for *P. tricornutum* in monoculture, and *r_dino_* and *K_dino_* for *P. minimum*. The interaction coefficient *α* (mean±SE) and C:N:P stoichiometry (particulate organic carbon to nitrogen (POC/PON) and particulate organic nitrogen to phosphorus (PON/POP); mol mol^-1^; mean±SE) are also shown for each treatment in bicultures.

|  | Low nutrient level | | | Normal nutrient level | | | High nutrient level | | |
| --- | --- | --- | --- | --- | --- | --- | --- | --- | --- |
|  | N:P = 10:1 | N:P = 24:1 | N:P = 63:1 | N:P = 10:1 | N:P = 24:1 | N:P = 63:1 | N:P = 10:1 | N:P = 24:1 | N:P = 63:1 |
| **12 ^o^C** |  |  |  |  |  |  |  |  |  |
| *μ_max_* | 0.64 | 0.65 | 0.51 | 0.84 | 0.94 | 1.06 | 0.75 | 0.95 | 1.02 |
| *μ* | 0.13 | 0.13 | 0.10 | 0.17 | 0.19 | 0.21 | 0.15 | 0.19 | 0.20 |
| *D* | 0.12 | 0.12 | 0.10 | 0.15 | 0.17 | 0.19 | 0.14 | 0.17 | 0.18 |
| *N_diatom0_* | 47.5±2.5 | 51.7±6.7 | 21.5±6.8 | 400.8±17.3 | 495.8±52.3 | 148.3±22.2 | 639.2±10.2 | 636.7±49.7 | 561.7±20.2 |
| *N_diatom1_* | 74.2±9.2 | 54.8±4.8 | 20.0±2.0 | 495.8±17.6 | 577.5±5.2 | 369.2±36.8 | 683.3±33.9 | 650.0±21.3 | 576.7±12.3 |
| *N_dino0_* | 1.7±0.3 | 1.7±0.8 | 2.2±1.2 | 1.2±0.2 | 0.7±0.4 | 1.3±0.4 | 1.2±0.3 | 1.3±0.3 | 2.2±0.8 |
| *N_dino1_* | 0.8±0.6 | 1.0±0.3 | 0.7±0.4 | 0.8±0.2 | 0.5±0.3 | 0.5±0.3 | 0.8±0.6 | 0.2±0.2 | 0.3±0.2 |
| *r_diatom_* | 1.15 | 0.65 | 1.01 | 0.92 | 0.93 | 0.83 | 0.87 | 0.98 | 1.03 |
| *r_dino_* | 0.37 | 0.26 | 0.61 | 0.28 | 0.45 | 0.93 | 0.89 | 0.55 | 0.28 |
| *K_diatom_* | 80.5 | 138.0 | 42.5 | 506.3 | 596.5 | 395.6 | 448.3 | 609.5 | 441.9 |
| *K_dino_* | 0.8 | 4.5 | 0.9 | 3.4 | 5.2 | 1.5 | 2.3 | 3.7 | 6.3 |
| *α* | 14.4±6.3 | 100.0±45.8 | 36.2±11.3 | 39.4±10.1 | 40.6±28.0 | 116.5±51.0 | -190.2±11.5 | -57.4±37.7 | -74.8±0.9 |
| POC/PON | 21.7±2.1 | 16.7±1.9 | 10.2±0.9 | 10.9±0.5 | 6.5±0.1 | 8.1±0.2 | 6.5±0.05 | 6.1±0.1 | 6.2±0.1 |
| PON/POP | 43.4±21.2 | 56.6±16.6 | 69.4 | 23.3±6.3 | 16.4±0.8 | 30.8±1.7 | 13.6±6.3 | 11.6±4.1 | 12.0±1.2 |
| **18 ^o^C** |  |  |  |  |  |  |  |  |  |
| *μ_max_* | 0.80 | 1.12 | 0.62 | 1.03 | 0.66 | 0.99 | 0.96 | 1.11 | 1.05 |
| *μ* | 0.16 | 0.22 | 0.12 | 0.21 | 0.13 | 0.20 | 0.19 | 0.22 | 0.21 |
| *D* | 0.15 | 0.20 | 0.12 | 0.19 | 0.12 | 0.18 | 0.18 | 0.20 | 0.19 |
| *N_diatom0_* | 79.2±0.8 | 100.0±8.7 | 47.5±2.9 | 493.3±5.8 | 462.5±3.8 | 290.8±7.3 | 669.2±8.8 | 721.7±5.1 | 514.2±5.1 |
| *N_diatom1_* | 75.0±3.8 | 124.2±7.9 | 15.0±0.8 | 627.5±12.3 | 720.8±13.0 | 533.3±15.3 | 613.3±25.3 | 556.7±3.0 | 531.3±13.8 |
| *N_dino0_* | 3.5±0.3 | 1.8±0.2 | 1.3±0.2 | 2.0±0.3 | 2.7±0.4 | 4.5±0.3 | 2.7±0.2 | 2.2±0.4 | 1.7±0.2 |
| *N_dino1_* | 0.7±0.2 | 1.0±0 | 1.5±0.3 | 0.3±0.3 | 1.8±0.3 | 0.5±0.3 | 0.5±0 | 0.3±0.2 | 0.3±0.3 |
| *r_diatom_* | 0.92 | 1.17 | 0.75 | 0.95 | 1.13 | 0.80 | 0.92 | 0.83 | 0.88 |
| *r_dino_* | 0.57 | 0.59 | 0.65 | 0.76 | 0.35 | 0.67 | 1.14 | 0.46 | 0.86 |
| *K_diatom_* | 93.3 | 163.2 | 53.8 | 497.2 | 591.7 | 391.4 | 477.2 | 646.5 | 386.4 |
| *K_dino_* | 0.9 | 5.9 | 0.9 | 2.6 | 27.6 | 2.6 | 2.1 | 24.9 | 2.8 |
| *α* | 8.4±1.7 | 34.0±5.8 | 23.8±2.7 | -78.0±20.3 | -13.1±4.4 | -21.9±7.1 | -100.4±4.2 | 26.6±7.0 | -136.5±5.2 |
| POC/PON | 19.9±0.5 | 16.1±0.4 | 10.5±0.6 | 11.2±0.2 | 6.7±0.1 | 8.0±0.1 | 6.6±0.03 | 6.7±0.1 | 6.3±0.2 |
| PON/POP | 12.8±0.5 | 53.6±14.9 | - | 13.6±2.3 | 54.4±20.2 | 88.9±7.6 | 4.8±0.2 | 10.0±4.9 | 17.1±4.2 |
| **24^o^C** |  |  |  |  |  |  |  |  |  |
| *μ_max_* | 0.41 | 0.66 | 0.36 | 1.13 | 1.13 | 1.09 | 1.07 | 1.20 | 1.02 |
| *μ* | 0.08 | 0.13 | 0.07 | 0.23 | 0.23 | 0.22 | 0.21 | 0.24 | 0.20 |
| *D* | 0.08 | 0.12 | 0.07 | 0.20 | 0.20 | 0.20 | 0.19 | 0.21 | 0.19 |
| *N_diatom0_* | 35.8±2.2 | 74.2±2.2 | 22.5±1.2 | 439.2±26.0 | 457.5±7.6 | 365.0±5.2 | 534.2±5.1 | 505.0±23.2 | 450.0±18.9 |
| *N_diatom1_* | 51.3±1.3 | 100.0±7.5 | 32.0±12.8 | 380.8±34.4 | 540.8±14.5 | 402.5±10.1 | 425.8±10.1 | 468.3±10.9 | 470.8±38.4 |
| *N_dino0_* | 3.8±0.6 | 2.2±0.3 | 1.0±0.5 | 2.5±0.3 | 6.3±0.6 | 4.0±0 | 3.8±0.2 | 3.8±0.6 | 3.5±0.6 |
| *N_dino1_* | 2.8±1.6 | 1.3±0.8 | 1.8±0.3 | 0 | 1.0±0.3 | 0.7±0.3 | 0.8±0.2 | 0.3±0.3 | 1.2±0.2 |
| *r_diatom_* | 1.47 | 0.58 | 0.99 | 1.43 | 1.18 | 1.53 | 1.60 | 0.83 | 1.40 |
| *r_dino_* | 0.65 | 0.43 | 0.37 | 0.57 | 0.64 | 0.73 | 0.79 | 0.57 | 0.41 |
| *K_diatom_* | 94.8 | 184.3 | 24.9 | 385.5 | 541.7 | 318.3 | 394.4 | 569.3 | 446.9 |
| *K_dino_* | 1.5 | 10.5 | 1.4 | 29.5 | 21.5 | 17.3 | 29.7 | 20.9 | 31.9 |
| *α* | 22.1±2.4 | 43.0±7.8 | 2.4±0.1 | -20.9±22.8 | 8.7±2.6 | -29.6±4.2 | -34.5±6.0 | 45.0±6.3 | -9.0±11.3 |
| POC/PON | 18.7±1.0 | 16.8±3.1 | 10.9±1.0 | 10.6±0.3 | 6.7±0.03 | 8.5±0.1 | 6.2±0.02 | 6.1±0.04 | 6.2±0.1 |
| PON/POP | 33.8 | - | 75.5 | 12.4±0.02 | 33.3±9.4 | 39.4±4.5 | 9.4±2.3 | 31.4±10.6 | 14.0±3.3 |

**Supplementary Table 2.** Results of Akaike information criterion corrected (AICc) in GLMMs for testing the effects of temperature, N:P supply ratios and nitrate concentrations on interaction coefficient α, POC/PON, PON/POP, 16:1/16:0, EPA/DHA and the ratios of brassicasterol/epi-brassicasterol to (brassicasterol/epi-brassicasterol + dinosterol) (B/(B + D)) in bicultures. The selected models are in bold. POC: particulate organic carbon; PON: particulate organic nitrogen; POP: particulate organic phosphorus; EPA: eicosapentaenoic acid (20:5n-3); DHA: docosahexaenoic acid (22:6n-3). The effect builder of ‘main’ refers to models containing first order effects of the three factors, with that of ‘main, two way’ for models containing first order effects and second order interactions and that of ‘main, two way and three way’ for models containing first order effects, second and third order interactions.

| Variable | Effect builder | AICc |
| --- | --- | --- |
| Interaction coefficient α | Main, two way and three way | 835.166 |
|  | Main, two way | 816.835 |
|  | **Main** | **803.190** |
| POC/PON | Main, two way and three way | 42.484 |
|  | Main, two way | 11.785 |
|  | **Main** | **-45.161** |
| PON/POP | Main, two way and three way | 145.609 |
|  | Main, two way | 118.778 |
|  | **Main** | **60.283** |
| 16:1/16:0 | Main, two way and three way | 36.101 |
|  | Main, two way | 33.416 |
|  | **Main** | **25.970** |
| EPA/DHA | Main, two way and three way | 440.900 |
|  | Main, two way | 417.449 |
|  | **Main** | **378.176** |
| B/(B + D) | Main, two way and three way | -134.275 |
|  | **Main, two way** | **-136.747** |
|  | Main | -137.953 |

**Supplementary Table 3.** The profiles of fatty acids (expressed as carbon-normalized content (*µ*g mg C^-1^) and percentages of total fatty acids (% of TFAs); mean ± SE) and sterols (expressed as *μ*g mg C^-1^; mean±SE) under different temperatures (12, 18 and 24 ^o^C), N:P supply ratios (molar ratios 10:1, 24:1 and 63:1) and nutrient concentrations (low, normal and high levels) in the bicultures of *Phaeodactylum tricornutum* and *Prorocentrum minimum*. SFAs, saturated fatty acids; MUFAs, monounsaturated fatty acids; PUFAs, polyunsaturated fatty acids; TFAs, total fatty acids.

|  | 12 ^o^C | | | | | | | | | | | | | | | | | |
| --- | --- | --- | --- | --- | --- | --- | --- | --- | --- | --- | --- | --- | --- | --- | --- | --- | --- | --- |
|  | Low nutrient level | | | | | | Normal nutrient level | | | | | | High nutrient level | | | | | |
|  | N:P = 10:1 | | N:P = 24:1 | | N:P = 63:1 | | N:P = 10:1 | | N:P = 24:1 | | N:P = 63:1 | | N:P = 10:1 | | N:P = 24:1 | | N:P = 63:1 | |
|  | Content | % | Content | % | Content | % | Content | % | Content | % | Content | % | Content | % | Content | % | Content | % |
| 14:0 | 39 | 6 | 46±7 | 8 | 18±0.4 | 6 | 45±4 | 8 | 18±4 | 8 | 31±2 | 10 | 14±1 | 7 | 14±2 | 7 | 15±6 | 8 |
| 16:0 | 255 | 39 | 241±37 | 44 | 124±11 | 37 | 159±10 | 29 | 43±9 | 21 | 95±6 | 30 | 37±3 | 18 | 35±4 | 18 | 33±12 | 19 |
| 16:1n-7 | 273 | 42 | 235±107 | 37 | 129±31 | 37 | 233±28 | 42 | 59±15 | 28 | 116±13 | 36 | 55±2 | 27 | 51±5 | 27 | 55±22 | 31 |
| 18:0 | 6 | 1 | 8±1 | 1 | 5±0.1 | 1 | 6±1 | 1 | 2±0.2 | 1 | 5±0.2 | 2 | 2±1 | 1 | 2±0.2 | 1 | 1±0.4 | 1 |
| 18:1n-9 | 39 | 6 | 25±9 | 4 | 24±5 | 7 | 12±4 | 2 | 10±3 | 4 | 8±1 | 2 | 11±1 | 5 | 10±2 | 5 | 7±3 | 4 |
| 18:2n-6 | 9 | 1 | 8±3 | 1 | 5±1 | 1 | 19±5 | 3 | 12±4 | 6 | 12±2 | 4 | 12±1 | 6 | 11±0.3 | 6 | 11±5 | 6 |
| 18:3n-6 | 3 | 0.4 | 2±1 | 0.3 | 2±1 | 1 | 2±1 | 0.3 | 1±0.2 | 0.3 | 1±0.1 | 0.2 | 0.5±0.03 | 0.2 | 0.3±0.04 | 0.1 | 0.4±0.2 | 0.2 |
| 20:4n-6 | 0 | 0 | 1±0 | 0.1 | 0.4±0.1 | 0.1 | 0.4±0.1 | 0.1 | 0.3±0.1 | 0.2 | 0.2±0.02 | 0.1 | 0.4±0.04 | 0.2 | 0.2±0.1 | 0.1 | 0.2±0.1 | 0.1 |
| 22:2n-6 | 1 | 0.1 | 1±1 | 0.2 | 1±0.2 | 0.2 | 1±1 | 0.2 | 1±0.3 | 0.4 | 2±0.3 | 0.6 | 1±0.1 | 0.7 | 1±0.1 | 0.5 | 0.2±0.1 | 0.2 |
| 24:0 | 2 | 0.3 | 2±0.1 | 0.3 | 1±0.05 | 0.3 | 3±1 | 0.5 | 4±0.02 | 2 | 4±0.3 | 1 | 4±0.4 | 2 | 4±1 | 2 | 3±0.3 | 2 |
| 20:5n-3 | 14 | 2 | 16±9 | 2 | 11±4 | 3 | 67±27 | 11 | 56±19 | 26 | 39±4 | 12 | 63±11 | 30 | 58±13 | 30 | 50±26 | 25 |
| 22:6n-3 | 3 | 0.5 | 2±1 | 0.3 | 17±6 | 5 | 8±3 | 1 | 5±2 | 2 | 4±0.5 | 1 | 5±1 | 2 | 4±1 | 2 | 5±2 | 2 |
| ∑SFAs^a^ | 303 | 47 | 298±46 | 54 | 149±12 | 45 | 214±13 | 39 | 67±13 | 32 | 137±8 | 43 | 57±5 | 28 | 55±6 | 29 | 53±19 | 31 |
| ∑MUFAs^b^ | 313 | 49 | 262±117 | 41 | 154±36 | 45 | 247±26 | 44 | 69±17 | 33 | 125±13 | 39 | 67±2 | 33 | 62±5 | 32 | 63±25 | 35 |
| ∑PUFAs^c^ | 29 | 5 | 31±14 | 5 | 38±13 | 11 | 99±36 | 17 | 76±25 | 35 | 58±6 | 18 | 82±13 | 39 | 75±14 | 39 | 67±34 | 34 |
| ∑TFAs | 646 |  | 590±177 |  | 340±61 |  | 560±73 |  | 213±55 |  | 319±27 |  | 207±8 |  | 193±10 |  | 183±78 |  |
| Brassicasterol/epi-brassicasterol | 4±1 |  | 4±2 |  | 2±1 |  | 6±1 |  | 6±0.3 |  | 4±1 |  | 5±0.2 |  | 5±0.3 |  | 7±0.5 |  |
| Dinosterol | 0.3±0.08 |  | 0.1±0.02 |  | 0.3±0.07 |  | 0.1 |  | 0.1 |  | 0 |  | 0.04 |  | 0 |  | 0 |  |

^a^ also includes 20:0 and 22:0 present at < 0.5% of TFAs in all treatments. ^b^ also includes 14:1, 20:1n-9, 22:1n-9 and 24:1n-9 present at < 0.5% of TFAs in all treatments. ^c^ also includes 18:3n-3, 20:2n-6, 20:3n-6 and 20:3n-3 present at < 0.5% of TFAs in all treatments.

**Supplementary Table 3.** Continued.

|  | 18 ^o^C | | | | | | | | | | | | | | | | | |
| --- | --- | --- | --- | --- | --- | --- | --- | --- | --- | --- | --- | --- | --- | --- | --- | --- | --- | --- |
|  | Low nutrient level | | | | | | Normal nutrient level | | | | | | High nutrient level | | | | | |
|  | N:P = 10:1 | | N:P = 24:1 | | N:P = 63:1 | | N:P = 10:1 | | N:P = 24:1 | | N:P = 63:1 | | N:P = 10:1 | | N:P = 24:1 | | N:P = 63:1 | |
|  | Content | % | Content | % | Content | % | Content | % | Content | % | Content | % | Content | % | Content | % | Content | % |
| 14:0 | 45±9 | 7 | 38±3 | 9 | 46±4 | 8 | 45±3 | 9 | 25±2 | 10 | 35±4 | 10 | 21±2 | 9 | 17±2 | 7 | 16±1 | 8 |
| 16:0 | 296±28 | 46 | 175±12 | 43 | 224±10 | 38 | 144±13 | 29 | 48±5 | 19 | 114±8 | 30 | 52±4 | 21 | 48±4 | 20 | 41±1 | 21 |
| 16:1n-7 | 220±46 | 33 | 149±30 | 35 | 194±27 | 33 | 211±26 | 42 | 70±8 | 28 | 156±14 | 41 | 72±7 | 30 | 66±7 | 28 | 54±2 | 28 |
| 18:0 | 12±2 | 2 | 7±0.4 | 2 | 9±0.1 | 1 | 6±0.3 | 1 | 2±0.1 | 1 | 6±0.4 | 2 | 2±0.1 | 1 | 2±0.1 | 1 | 2±0.01 | 1 |
| 18:1n-9 | 55±8 | 8 | 18±3 | 4 | 66±5 | 11 | 10±2 | 2 | 20±3 | 8 | 10±1 | 2 | 19±3 | 8 | 22±4 | 9 | 13±1 | 7 |
| 18:2n-6 | 6±1 | 1 | 6±1 | 2 | 7±1 | 1 | 15±3 | 3 | 9±2 | 4 | 9±1 | 2 | 10±1 | 4 | 10±1 | 4 | 8±0.3 | 4 |
| 18:3n-6 | 2±0.3 | 0.2 | 1±0.4 | 0.3 | 3±1 | 0.5 | 2±1 | 0.4 | 1±0.1 | 0.4 | 1±0.2 | 0.3 | 1±0.2 | 0.3 | 1±0.1 | 0.3 | 1±0.04 | 0.3 |
| 20:4n-6 | 0±0 | 0 | 0.5±0.04 | 0.1 | 1±0.1 | 0.1 | 1±0.2 | 0.1 | 1±0.1 | 0.4 | 0.4±0.1 | 0.1 | 1±0.2 | 0.3 | 1±0.2 | 0.4 | 1±0.1 | 0.3 |
| 22:2n-6 | 0.4±0.04 | 0.1 | 1±0.3 | 0.3 | 1±0.3 | 0.2 | 1±0.4 | 0.2 | 1±0.2 | 0.4 | 3±0.4 | 1 | 1±0.3 | 1 | 1±0.2 | 0.4 | 1±0.1 | 1 |
| 24:0 | 2±0.1 | 0.3 | 2±0.2 | 1 | 1±0.04 | 0.2 | 3±0.5 | 1 | 4±0.4 | 2 | 4±0.2 | 1 | 4±0.3 | 1 | 3±0.3 | 1 | 4±0.2 | 2 |
| 20:5n-3 | 9±2 | 1 | 12±4 | 3 | 12±4 | 2 | 60±20 | 11 | 61±12 | 24 | 38±7 | 9 | 57±13 | 23 | 64±14 | 26 | 49±8 | 25 |
| 22:6n-3 | 4±0.2 | 1 | 1±0.3 | 0.3 | 16±3 | 3 | 7±2 | 1 | 4±1 | 2 | 3±0.4 | 1 | 3±1 | 1 | 3±1 | 1 | 4±1 | 2 |
| ∑SFAs^a^ | 357±34 | 55 | 224±16 | 55 | 282±14 | 48 | 200±16 | 40 | 80±6 | 32 | 160±12 | 42 | 80±6 | 33 | 71±6 | 30 | 65±1 | 33 |
| ∑MUFAs^b^ | 277±55 | 42 | 167±33 | 40 | 261±32 | 44 | 222±28 | 44 | 90±11 | 36 | 167±15 | 44 | 92±10 | 38 | 89±11 | 37 | 68±3 | 35 |
| ∑PUFAs^c^ | 22±3 | 3 | 23±5 | 5 | 49±17 | 8 | 86±26 | 16 | 79±15 | 31 | 55±9 | 14 | 74±16 | 30 | 81±16 | 33 | 64±9 | 32 |
| ∑TFAs | 656±92 |  | 414±54 |  | 593±64 |  | 509±70 |  | 248±33 |  | 382±36 |  | 245±32 |  | 241±33 |  | 197±13 |  |
| Brassicasterol/epi-brassicasterol | 3±0.4 |  | 2±1 |  | 2±0.3 |  | 8±0.4 |  | 6±0.4 |  | 5±0.2 |  | 5±0.2 |  | 6±1 |  | 5±1 |  |
| Dinosterol | 0.6±0.01 |  | 0.1±0.01 |  | 0.8±0.04 |  | 0.1±0.01 |  | 0.1±0.01 |  | 0.1±0.002 |  | 0.1 |  | 0.04 |  | 0.06±0.01 |  |

**Supplementary Table 3.** Continued.

|  | 24^o^C | | | | | | | | | | | | | | | | | |
| --- | --- | --- | --- | --- | --- | --- | --- | --- | --- | --- | --- | --- | --- | --- | --- | --- | --- | --- |
|  | Low nutrient level | | | | | | Normal nutrient level | | | | | | High nutrient level | | | | | |
|  | N:P = 10:1 | | N:P = 24:1 | | N:P = 63:1 | | N:P = 10:1 | | N:P = 24:1 | | N:P = 63:1 | | N:P = 10:1 | | N:P = 24:1 | | N:P = 63:1 | |
|  | Content | % | Content | % | Content | % | Content | % | Content | % | Content | % | Content | % | Content | % | Content | % |
| 14:0 | 45±1 | 6 | 29±8 | 6 | 27±5 | 6 | 26±7 | 7 | 18±3 | 8 | 23±2 | 7 | 20±2 | 7 | 18±3 | 8 | 20±3 | 7 |
| 16:0 | 323±33 | 44 | 179±55 | 38 | 191±46 | 44 | 102±22 | 29 | 49±7 | 21 | 94±10 | 29 | 55±0.4 | 20 | 48±3 | 21 | 62±2 | 22 |
| 16:1n-7 | 263±47 | 35 | 178±43 | 39 | 132±33 | 30 | 143±27 | 40 | 67±6 | 29 | 131±17 | 39 | 77±4 | 28 | 64±7 | 28 | 82±6 | 29 |
| 18:0 | 13±1 | 2 | 10±4 | 2 | 9±2 | 2 | 4±1 | 1 | 2±0.4 | 1 | 5±0.4 | 2 | 3±1 | 1 | 3±0.3 | 1 | 4±1 | 2 |
| 18:1n-9 | 62±4 | 8 | 29±12 | 6 | 55±20 | 12 | 12±1 | 3 | 16±0.4 | 7 | 13±5 | 4 | 25±3 | 9 | 21±4 | 9 | 23±3 | 8 |
| 18:2n-6 | 6±2 | 1 | 5±0.1 | 1 | 5±1 | 1 | 7±1 | 2 | 9±0.1 | 4 | 8±2 | 2 | 11±1 | 4 | 9±1 | 4 | 12±1 | 4 |
| 18:3n-6 | 3±1 | 0.4 | 3±1 | 1 | 2±1 | 0.4 | 2±0.1 | 1 | 1±0.1 | 1 | 3±1 | 1 | 1±0.1 | 0.4 | 1±0.2 | 0.5 | 1±0.2 | 0.5 |
| 20:4n-6 | 1±0.4 | 0.1 | 2±1 | 0.4 | 1±0.2 | 0.1 | 2±0.1 | 0.5 | 2±0.2 | 1 | 1±1 | 0.4 | 2±0.3 | 1 | 2±0.3 | 1 | 2±0.4 | 1 |
| 22:2n-6 | 1±1 | 0.1 | 1±0.4 | 0.3 | 1±0.2 | 0.2 | 1±0.04 | 0.3 | 1±0.2 | 1 | 4±1 | 1 | 1±0.1 | 0.3 | 1±0.1 | 0.3 | 1±0.2 | 0.3 |
| 24:0 | 2±0.3 | 0.3 | 1±0.1 | 0.3 | 1±0.1 | 0.2 | 3±0.5 | 1 | 3±0.2 | 1 | 2±0.04 | 1 | 3±0.3 | 1 | 3±0.04 | 1 | 4±0.3 | 1 |
| 20:5n-3 | 22±15 | 3 | 17±3 | 5 | 7±2 | 2 | 42±6 | 13 | 54±13 | 24 | 45±18 | 13 | 69±14 | 25 | 57±14 | 24 | 68±15 | 23 |
| 22:6n-3 | 4±1 | 1 | 2±0.3 | 1 | 7±2 | 2 | 5±0.4 | 1 | 3±1 | 2 | 3±1 | 1 | 4±1 | 1 | 3±1 | 1 | 4±0.5 | 1 |
| ∑SFAs^a^ | 385±35 | 52 | 220±67 | 47 | 229±53 | 53 | 136±31 | 38 | 73±10 | 32 | 126±12 | 38 | 82±1 | 30 | 72±6 | 32 | 91±3 | 32 |
| ∑MUFAs^b^ | 326±48 | 43 | 208±55 | 45 | 187±53 | 42 | 155±28 | 44 | 83±6 | 36 | 145±22 | 43 | 102±7 | 37 | 85±10 | 37 | 106±9 | 37 |
| ∑PUFAs^c^ | 38±20 | 5 | 31±3 | 8 | 23±5 | 5 | 59±5 | 18 | 72±14 | 32 | 66±25 | 19 | 89±16 | 32 | 73±17 | 31 | 90±18 | 31 |
| ∑TFAs | 748±82 |  | 460±120 |  | 439±111 |  | 351±54 |  | 228±3 |  | 337±59 |  | 274±25 |  | 231±33 |  | 287±30 |  |
| Brassicasterol/epi-brassicasterol | 3±1 |  | 3±1 |  | 2±0.2 |  | 7±1 |  | 6±1 |  | 7±1 |  | 6±0.3 |  | 5±0.4 |  | 7±1 |  |
| Dinosterol | 1.3±0.3 |  | 0.5±0.1 |  | 1.3±0.3 |  | 0.2±0.03 |  | 0.2±0.05 |  | 0.3±0.04 |  | 0.1±0.01 |  | 0.1 |  | 0.2±0.02 |  |
